# Identification of multiple odorant receptors essential for pyrethrum repellency in *Drosophila melanogaster*

**DOI:** 10.1371/journal.pgen.1009677

**Published:** 2021-07-08

**Authors:** Qiang Wang, Peng Xu, Felipe Andreazza, Yahui Liu, Yoshiko Nomura, Phil Duran, Lan Jiang, Mengli Chen, Genki Takamatsu, Makoto Ihara, Kazuhiko Matsuda, Rufus Isaacs, Eugenio E. Oliveira, Yuzhe Du, Ke Dong

**Affiliations:** 1 Department of Entomology, Michigan State University, East Lansing, Michigan, United States of America; 2 Department of Preventive Medicine and Public Health Laboratory Science, School of Medicine, Jiangsu University, Zhenjiang, China; 3 Department of Entomology, Universidade Federal de Viçosa, Viçosa, Brazil; 4 Department of Biology, Duke University, Durham, North Carolina, United States of America; 5 Department of Plant Protection, School of Agriculture and Food Science, Zhejiang Agriculture and Forestry University, Hangzhou, China; 6 Department of Biological Sciences, Oakland University, Rochester, Michigan, United States of America; 7 Institute of Pesticide and Environmental Toxicology, Zhejiang University, Hangzhou, China; 8 Department of Applied Biological Chemistry, Faculty of Agriculture, Kindai University, Nara, Japan; 9 Agricultural Technology and Innovation Research Institute, Kindai University, Nara, Japan; 10 Ecology, Evolutionary Biology, and Behavior Program, Michigan State University, East Lansing, Michigan, United States of America; University of Kentucky, UNITED STATES

## Abstract

Pyrethrum extract from dry flowers of *Tanacetum cinerariifolium* (formally *Chrysanthemum cinerariifolium*) has been used globally as a popular insect repellent against arthropod pests for thousands of years. However, the mechanistic basis of pyrethrum repellency remains unknown. In this study, we found that pyrethrum spatially repels and activates olfactory responses in *Drosophila melanogaster*, a genetically tractable model insect, and the closely-related *D*. *suzukii* which is a serious invasive fruit crop pest. The discovery of spatial pyrethrum repellency and olfactory response to pyrethrum in *D*. *melanogaster* facilitated our identification of four odorant receptors, Or7a, Or42b, Or59b and Or98a that are responsive to pyrethrum. Further analysis showed that the first three Ors are activated by pyrethrins, the major insecticidal components in pyrethrum, whereas Or98a is activated by (*E*)-β-farnesene (EBF), a sesquiterpene and a minor component in pyrethrum. Importantly, knockout of *Or7a*, *Or59b* or *Or98a* individually abolished fly avoidance to pyrethrum, while knockout of *Or42b* had no effect, demonstrating that simultaneous activation of Or7a, Or59b and Or98a is required for pyrethrum repellency in *D*. *melanogaster*. Our study provides insights into the molecular basis of repellency of one of the most ancient and globally used insect repellents. Identification of pyrethrum-responsive Ors opens the door to develop new synthetic insect repellent mixtures that are highly effective and broad-spectrum.

## Author summary

Pyrethrum extract began to be used as an insect repellent against biting arthropods and blood-sucking mosquitoes since ancient times. However, the mechanisms underlying pyrethrum repellency remains unknown. In this study, we took advantage of *Drosophila melanogaster* as a model insect system for olfaction studies and conducted a series of electrophysiological, molecular genetic and behavioral experiments to understand the mechanism of pyrethrum repellency in *D*. *melanogaster*. We discovered that pyrethrum repels *D*. *melanogaster* by activating multiple odorant receptors (Ors). Apparently simultaneous activation of these Ors by various components in pyrethrum extract makes pyrethrum one of the most potent and the longest used insect repellents in the human history.

## Introduction

Pyrethrum is a botanical insecticide extracted from dry flowers of *Tanacetum cinerariifolium* (also known as *Chrysanthemum cinerariifolium*). This plant is grown commercially in many parts of the world, particularly in East Africa and Australia, for extraction of pyrethrum, which accumulates in the flower achenes [1,2]. Pyrethrum is non-persistent in the environment and possesses low mammalian toxicity. Pyrethrum extract contains three structurally closely-related insecticidal esters of chrysanthemic acid (pyrethrin I) and three corresponding esters of pyrethric acid (pyrethrins II). Pyrethrins are prototypes of pyrethroids, a large class of widely used synthetic insecticides [3]. Pyrethrins and pyrethroids target voltage-gated sodium channels for their insecticidal effects [4–6], which is critical for the initiation and propagation of action potentials in the nervous system. Pyrethrins and pyrethroids promote activation of sodium channels and inhibit deactivation and inactivation, which lead to the disruption of the function of the nervous system.

Besides the insecticidal activities, pyrethrum extract has also been used as an insect repellent against biting arthropods for thousands of years [7] and in mosquito coils for more than a century [8]. In addition, pyrethrum-producing *Chrysanthemum* spp. are recommended as companion plants to repel pest insects [9]. Recent studies experimentally demonstrated behavioral deterrence of pyrethrin-containing *Chrysanthemum* leaves against western flower thrips (*Frankliniella occidentalis*) [10] and spatial repellency of a pyrethrin precursor against cotton aphids (*Aphis gossypii*) [11]. Despite these studies, the mechanistic basis of pyrethrum repellency remains unknown until our recent study in *Aedes aegypti* [12] and this study in *Drosophila melanogaster*.

*Drosophila melanogaster* has been an excellent model for studying insect olfactory chemosensing, with distinct types of well-characterized olfactory receptor neurons (ORNs) [13–20]. ORNs are housed in hair-like olfactory sensilla on the antennae. With a few exceptions, each sensillum usually houses two (up to four) ORNs and each ORN expresses one specific odorant receptor (Or) protein. Activation of Ors by odorants excites ORNs which project axons to the antennal lobe in the brain, where signals are processed and transmitted to higher order centers, which triggers appropriate behavioral outcomes.

In this study, we discovered that pyrethrum activate antennal olfactory receptor neurons and elicit spatial repellency in *D*. *melanogaster*, a model insect, as well as *D*. *suzukii*, a serious global insect pest of economically valuable small fruit and tree fruit crops [21]. We then further investigated the underlying mechanism of pyrethrum repellency by taking advantage of *D*. *melanogaster* as a model for olfactory studies. We found that specific components of pyrethrum activate multiple odorant receptors (Ors) and that co-activation of these Ors are essential for pyrethrum repellency. Identification of pyrethrum-responsive Ors represents a major step forward in the understanding of the molecular basis of repellency of one of the most ancient and globally used insect repellents.

## Results

### Pyrethrum repels *D*. *melanogaster* and *D*. *suzukii*

**Fig 1:**
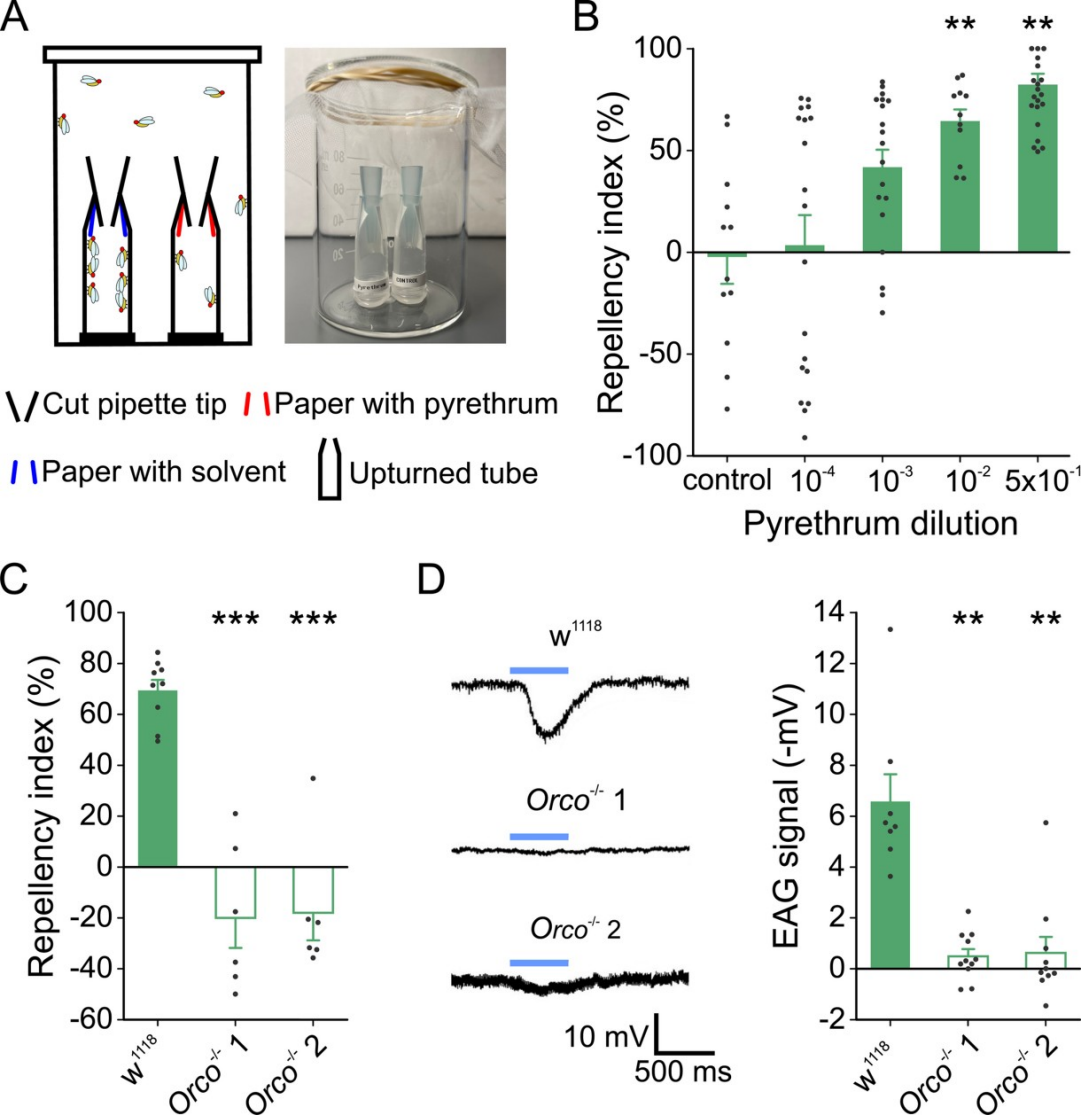
*Drosophila melanogaster* shows odorant receptor-dependent avoidance response to pyrethrum. (A) Schematic drawing of a two-choice assay. (B) Behavioral responses to pyrethrum delivered in 50 μL of various dilutions (v v^-1^) in a two-choice assay (*H* = 32.15, *d*.*f*. = 4, *P* < 0.001; **P < 0.01 compared to control; One-Way ANOVA on Ranks, *n* = 11 for 10^−2^, *n* = 12 for control, n = 19 for 10^−4^ and 10^−3^, and *n* = 20 for 0.5 dilution). (C) Orco-dependency of repellency to pyrethrum at 50 μL of the 0.5 dilution (v v^-1^) (*F*_(2,18)_ = 40.60, *P* < 0.001; ***P < 0.001 compared to w^1118^; One-Way ANOVA, *n* = 9 for w^1118^, and *n* = 6 for the rest). (D) Orco-dependency of EAG responses to pyrethrum at 30 μL of the 10^−2^ dilution (v v^-1^) (*H* = 15.07, *d*.*f*. = 2, *P* < 0.001; **P < 0.01 compared to w^1118^; One-Way ANOVA on Ranks, *n* = 8 for w^1118^, *n* = 11 for *Orco*^-/-^ 1, and *n* = 10 for *Orco*^-/-^ 2).

To evaluate whether pyrethrum repels *D*. *melanogaster*, we first used a two-choice assay (Fig 1A) that is similar to that described previously [22]. We found that pyrethrum repelled *D*. *melanogaster* w^1118^ adults at the 10^−2^ dilution (v v^-1^) (Fig 1B). The avoidance behavior was also observed in a T-maze assay (S1 Fig) which was modified from a previously reported protocol [22,23]. Pyrethrum also repelled *D*. *suzukii* in these assays (S1 Fig). Furthermore, we performed the two-choice assay in the presence of an attractant, apple cider vinegar (ACV), i.e., a two-choice attraction assay [24] (S1 Fig) and found that both *D*. *melanogaster* and *D*. *suzukii* were repelled by pyrethrum in this assay as well (S1 Fig).

### Orco-dependent pyrethrum-avoidance behavior of *D*. *melanogaster*

Perception of volatile chemicals by insects begins when the volatiles enter the lymph of olfactory sensilla and activate Ors or ionotropic receptors (Irs) located on the dendritic surface of olfactory receptor neurons (ORNs) [14,17,25]. Individual ORNs of basiconic and trichoid sensilla each express a single member of the Or family, which confers a characteristic odorant response profile of that neuron [26]. Each Or is co-expressed with an obligate olfactory receptor co-receptor (Orco) [27], which is essential for odorant perception [28,29]. To determine whether the avoidance behavior to pyrethrum we observed is Orco-dependent, we examined the behavioral response of olfactory defective *Orco* mutant flies [29] to pyrethrum. We found avoidance behavior to pyrethrum was completely abolished in two *Orco* mutant lines (Fig 1C) and the RI of *Orco* mutants to pyrethrum was not significantly different from that of solvent control (*F*_(2,15)_ = 1.02, *P* = 0.39; One-Way ANOVA, *n* = 6). Consistent with this finding, pyrethrum elicited robust olfactory signals in response to pyrethrum in electroantennography (EAG) recording of antennae of adult *D*. *melanogaster*, but no such EAG signals were detected in antennae of the two *Orco* mutant lines (Fig 1D).

### Electrophysiological responses of ORNs to pyrethrum in *D*. *melanogaster* and *D*. *suzukii*

**Fig 2:**
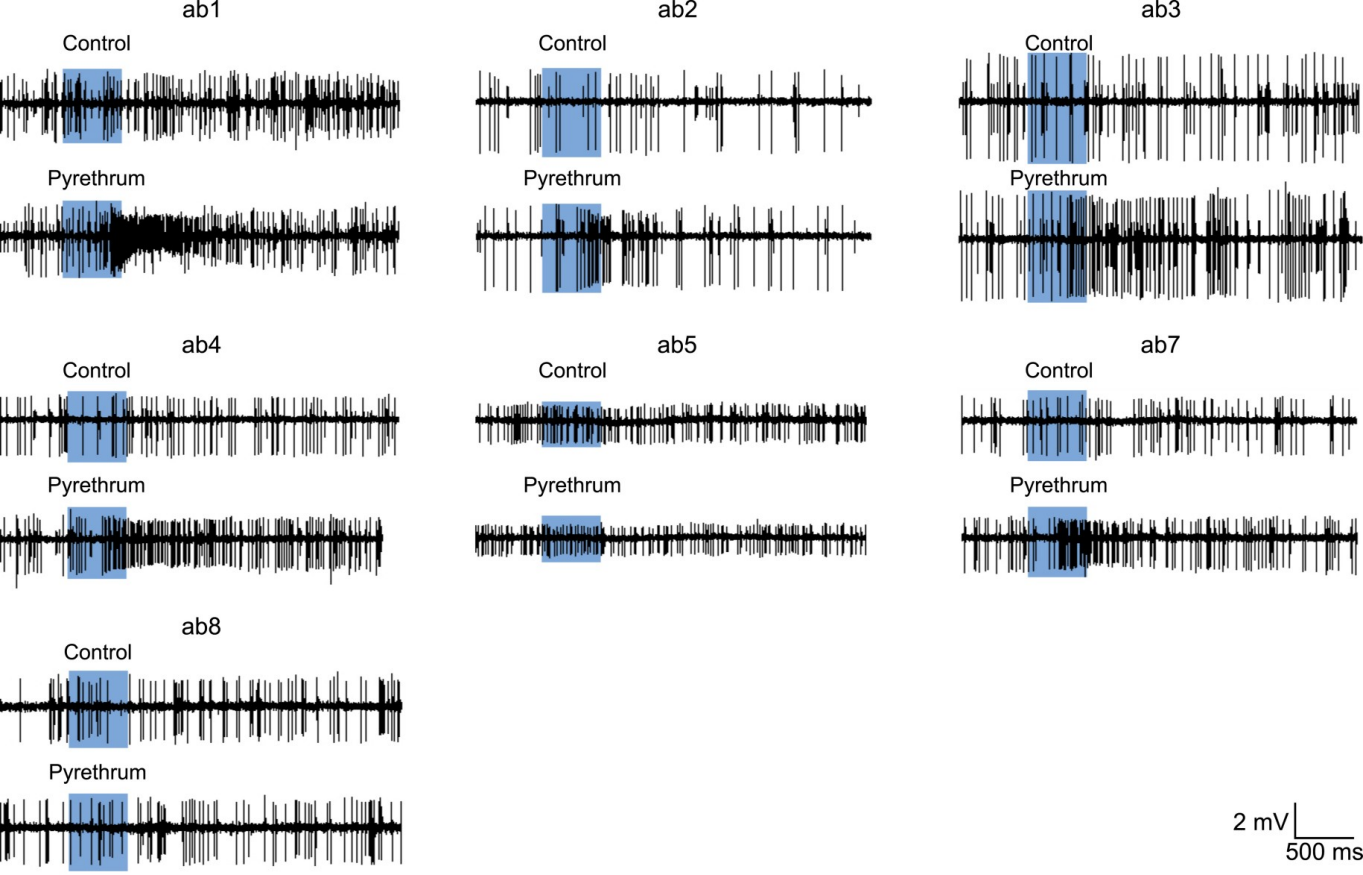
Single sensillum recording of odorant receptor neurons to pyrethrum in *D*. *melanogaster*. Representative SSR traces showing ORN responses to pyrethrum at 30 μL of the 10^−2^ dilution (v v^-1^) in ab1, ab2, ab3, ab4, ab5, ab7, and ab8 sensilla.

To identify which ORNs respond to pyrethrum, we focused on ORNs housed in antennal basiconic (ab) sensilla, where most antennal Orco/Ors are expressed [30]. Except for ab1, which contains four neurons, all ab sensilla house two neurons. We conducted single sensillum recording (SSR) of the electrical activities (i.e., action potentials measured as spikes/second) of ORNs in ab sensilla, as described by de Bruyne *et al*. [13]. Neurons that generate larger spikes in response to odors are defined as A neurons, whereas neurons that produce smaller spikes are called B neurons. We first recorded SSR responses to a panel of standard discriminating odorants [31,32] to ensure accurate identification and normalcy of each sensillum. Using this method, we were able to locate ab1-5 and ab7-8 sensilla (S2 Fig). We then examined the response of ORNs in ab1-5 and ab7-8 sensilla to pyrethrum. Representative traces of SSR measurements from ab1-5 and ab7-8 are presented in Fig 2. Pyrethrum increased the firing frequency of five out of 16 neurons in ab1, ab2, ab3, ab4 and ab7 sensilla (Table 1). In contrast, pyrethrum did not activate any neurons in ab5 or ab8 sensilla.

**Table 1:** Response spectra of ORNs to pyrethrum in *D*. *melanogaster* and *D*. *suzukii*.

Species	Antenna basiconic sensilla															
	ab1				ab2		ab3		ab4		ab5		ab7		ab8	
	A	B	C	D	A	B	A	B	A	B	A	B	A	B	A	B
*D*. *melanogaster*	+	●	●	●	+	●	+	●	+	●	●	●	+	●	●	●
*D*. *suzukii*	+	●	●	●	+	++	+	●	+	●	●	●	+	●	●	●

Note: “●,” n < 20 spikes/s; “+,” 20 ≤ n < 40 spikes/s; “++,” n ≥ 40 spikes/s. Each compound was delivered in 30 μL of the 10^−2^ dilution (v v^-1^); *n* = 6 flies/sensilla.

Using the same panel of discriminating odorants, we identified ab1-5 and ab7-8 sensilla in the antennae of *D*. *suzukii* (S3 Fig). Interestingly, the response profiles to standard discriminating odorants were essentially identical to those in *D*. *melanogaster* with one exception. In *D*. *suzukii*, ab2B neurons displayed strong responses to 2-heptanone, which was not observed in *D*. *melanogaster*, as also reported by Keesey *et al*. [31]. As in *D*. *melanogaster*, pyrethrum activated neurons of ab1A, ab2A, ab3A, ab4A and ab7A of *D*. *suzukii* (S4 Fig and Table 1). In addition, ab2B neuron of *D*. *suzukii* responded to pyrethrum, which was not seen in *D*. *melanogaster* (Table 1).

### Identification of Ors activated by pyrethrum

**Fig 3:**
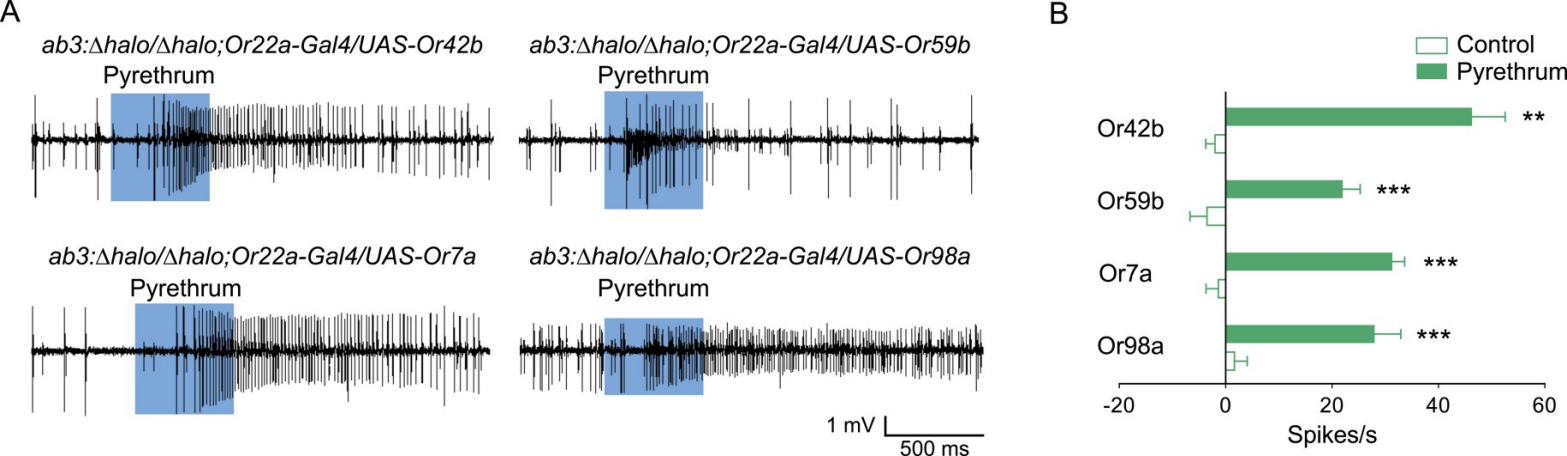
Pyrethrum responses of four *D*. *melanogaster* odorant receptors in the ab3A empty neuron system. (A) Representative SSR traces showing responses to pyrethrum at 30 μL of the 10^−1^ dilution (v v^-1^) of Or42b, Or59b, Or7a, and Or98a expressed in the ab3A empty neuron (*ab3*: *Δhalo/Δhalo; Or22a-Gal4/UAS-Orx*). (B) Responses to pyrethrum at 30 μL of the 10^−1^ dilution (v v^-1^) of ab3A neurons expressing Or7a (*t* = -9.84, *d*.*f*. = 10, ***P < 0.001 compared to control, *t*-test, *n* = 6), Or42b (*U* = 0.0, **P < 0.01 compared to control, Mann-Whitney rank sum test, *n* = 6), Or59b (*t* = -5.47, *d*.*f*. = 12, ***P < 0.001 compared to control, *t*-test, *n* = 8 for control, and *n* = 6 for pyrethrum) and Or98a (*t* = -5.35, *d*.*f*. = 8, ***P < 0.001 compared to control, *t*-test, *n* = 6 for control, and *n* = 4 for pyrethrum).

Maps of *Or* gene expression in basiconic sensilla are well established in the *D*. *melanogaster* olfactory system [30]. To identify which Ors are activated by pyrethrum, we employed the ab3 “empty neuron” system [33] by genetically introducing Ors, individually, into the A neurons of the empty ab3 sensilla, in which its endogenous *Or* gene Or22a is deleted. SSR analysis of the recombinant ab3 sensilla expressing each of the heterologously introduced Ors confirmed that Or42b from ab1A, Or59b from ab2A, Or7a from ab4A, and Or98a from ab7A were activated by pyrethrum (Fig 3A and 3B). Since Or22a is expressed in ab3A, we cannot directly test the role of Or22a in sensing pyrethrum in the empty neuron system. Consistent with SSR results, Ors in pyrethrum-nonresponsive ab1D, ab2B, ab5A, ab5B, ab7B and ab8A/B neurons could not be activated by pyrethrum in the empty neuron system (Table 2). In addition, we also examined Or49b from ab6B, and Or67a and Or85f from ab10A and ab10B sensilla, respectively, in the empty neuron system because we could not directly identify these two types of sensilla in SSR. We found that none of them were activated by pyrethrum (Table 2). Taken together, our results showed that four Ors, Or7a, Or42b, Or59b and Or98a, are activated by pyrethrum in *D*. *melanogaster*.

**Table 2:** Responses to pyrethrum of 16 odorant receptors in the *Drosophila* ab3A empty neuron.

dilution	ab1		ab2		ab4	ab5			ab6	ab7		ab8		ab10	
	42b	10a	59b	85a	7a	82a	33b	47a	49b	98a	67c	43b	9a	67a	85f
10^−2^	++	●	●	●	+	●	●	●	●	●	●	●	●	●	●
10^−1^	+++	●	+	●	++	●	●	●	●	+	●	●	●	●	●

Note: “●,” n < 20 spikes/s; “+,” 20 ≤ n < 40 spikes/s; “++,” 40 ≤ n < 60 spikes/s; “+++,” n ≥ 60 spikes/s. Each compound was delivered in 30 μL of the 10^−2^ dilution (v v^-1^). *n* = 6 flies/sensilla. The original sensilla (ab1 –ab10) in which 16 Ors are expressed are indicated above Ors.

### Selective activation of pyrethrum-responsive Ors by different components in pyrethrum

Pyrethrum extract contains six structurally related esters: pyrethrin I, cinerin I and jasmolin I, which are three esters of chrysanthemic acid, and pyrethrin II, cinerin II and jasmolin II, which are esters of pyrethric acid (S5 Fig). The structures of these compounds differ only in the acid and alcohol termini. Pyrethrin I and pyrethrin II are predominant components (together constituting more than 50%) in pyrethrum extracts [1,3]. We tested the effects of the six individual compounds on the pyrethrum-responsive Ors expressed in the ab3 empty neuron system (Table 3). Or42b was activated by multiple components including pyrethrin II, jasmolin I and II (Table 3 and S5 Fig). Or7a was activated by pyrethrin I and II (Table 3 and S5 Fig). Or59b was activated by pyrethrin II and jasmolin II (Table 3 and S5 Fig).

**Table 3:** Responses to pyrethrum components of four pyrethrum-responsive odorant receptors in the *Drosophila* ab3A empty neuron.

Components	Or42b	Or7a	Or59b	Or98a
Pyrethrin I	+	+++	●	●
Pyrethrin II	+++	++	+	●
Jasmolin I	+++	●	●	●
Jasmolin II	+++	●	+	●
Cinerin I	+	●	●	●
Cinerin II	●	●	●	●
(*E*)-β-farnesene	●	●	●	+
Control	●	●	●	●

Note: “●,” n < 20 spikes/s; “+,” 20 ≤ n < 40 spikes/s; “++,” 40 ≤ n < 60 spikes/s; “+++,” n ≥ 60 spikes/s. Each compound was delivered in 30 μL of 100 mmol L^-1^ except for (*E*)-β-farnesene which was delivered in 30 μL of 394 mmol L^-1^; n = 6 flies/sensilla.

None of the six pyrethrin components activated Or98a (Table 3), suggesting another compound, likely minor in the pyrethrum extract, activates Or98a. (*E*)-β-farnesene (EBF) (S5 Fig) and several other phytoterpenes, are found as minor components in pyrethrum extracts and contribute to the flowery fragrance of pyrethrum extract [3,34]. Therefore, we next tested if EBF could activate Or98a. As shown in Table 3 and S5 Fig, EBF activated Or98a, but not other pyrethrum-responsive Ors. Taken together, our functional analysis in the *D*. *melanogaster* empty neuron system revealed that the major components of pyrethrum activate Or7a, Or42b, and/or Or59b; whereas a minor component, EBF, activates Or98a.

### Effect of knockout of *Or7a*, *Or42b*, *Or59b* and *Or98a* on fly aversion to pyrethrum

**Fig 4:**
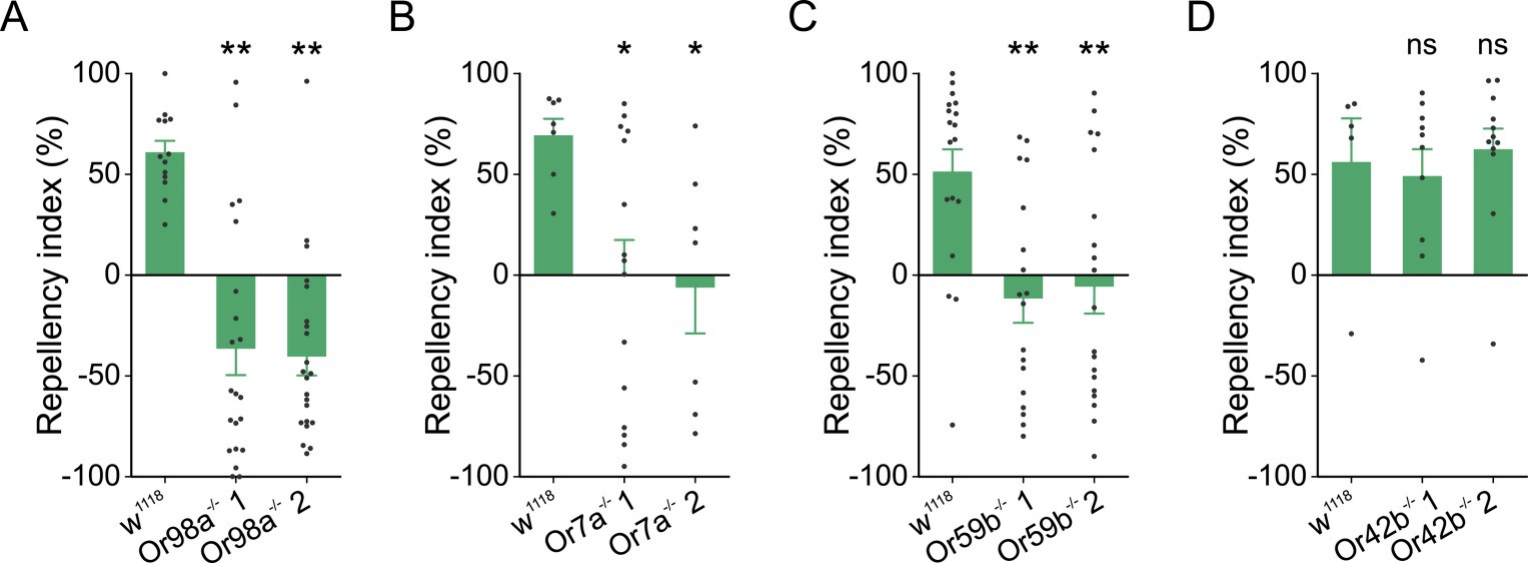
Knockout of *Or7a*, *Or59b* and *Or98a*, but not of *Or42b*, abolished fly aversion to pyrethrum. (A) Behavioral responses of w^1118^ and two Or98a^-/-^ lines to pyrethrum at 50 μL of the 10^−2^ dilution (v v^-1^) (*H* = 21.80, *d*.*f*. = 2, *P* < 0.001; **P < 0.01 compared to w^1118^, One-Way ANOVA on Ranks, *n* = 13 for w^1118^, *n* = 21 for Or98a^-/-^ 1, and *n* = 22 for Or98a^-/-^ 2). (B) Behavioral responses of w^1118^ and two Or7a^-/-^ lines to pyrethrum at 50 μL of the 10^−2^ dilution (v v^-1^) (*H* = 8.16, *d*.*f*. = 2, *P* = 0.017; *P < 0.05 compared to w^1118^, One-Way ANOVA on Ranks, *n* = 7 for w^1118^, *n* = 15 for Or7a^-/-^ 1, and *n* = 7 for Or7a^-/-^ 2). (C) Behavior responses of w^1118^ and two Or59b^-/-^ lines to pyrethrum at 50 μL of the 10^−2^ dilution (v v^-1^) (*F*_(2,52)_ = 7.92, *P* < 0.001; **P < 0.01 compared to w^1118^, One-Way ANOVA, *n* = 18 for w^1118^ and for Or59b^-/-^ 1, and *n* = 19 for Or59b^-/-^ 2). (D) Behavioral responses of w^1118^ and two Or42b^-/-^ lines to pyrethrum at 50 μL of the 10^−2^ dilution (v v^-1^) (*H* = 0.47, *d*.*f*. = 2, *P* = 0.79; ns = not significant compared to w^1118^, One-Way ANOVA on Ranks, *n* = 5 for w^1118^, *n* = 10 for Or42b^-/-^ 1, and *n* = 12 for Or42b^-/-^ 2).

Identification of *D*. *melanogaster* Ors that are activated by specific components in pyrethrum provides a foundation for functional dissection of the molecular basis of avoidance behavior to pyrethrum. We knocked out *Or7a*, *Or42b*, *Or59b* and *Or98a* individually in *D*. *melanogaster* using the CRISPR-Cas9 technology and generated two independent knockout lines for each gene (S6 Fig). Pyrethrum repellency was completely abolished in *Or7a*^-/-^, *Or59b*^-/-^ and *Or98a*^-/-^ lines (Fig 4A-C), but not in two *Or42b*^-/-^ lines (Fig 4D). Furthermore, *Or98a*^-/-^ flies not only lost aversion response to pyrethrum, but also displayed significant attraction to pyrethrum (Fig 4A). These results indicate that simultaneous activation of Or7a, Or59b and Or98a is essential for fly avoidance to pyrethrum, as knockout of any of the three Ors abolishes pyrethrum repellency.

## Discussion

In this study, we discovered that pyrethrum vapor evokes olfactory responses and elicits aversion in *D*. *melanogaster* and *D*. *suzukii*. Although the major components of pyrethrum, pyrethrins, are known to target voltage-gated sodium channels for their insecticidal activity [35], *we* show here that pyrethrins also activate three Ors, Or7a, Or42b and Or59b. In addition, we discovered that EBF, a minor component in pyrethrum, activates another Or, Or98a. The most intriguing discovery of this study is that three Ors, Or7a, Or59b and Or98a, that are activated by multiple components in pyrethrum are all essential for pyrethrum repellency. Our results provide insights into the molecular basis of repellency of one of the most ancient and globally used insect repellents. It appears that simultaneous activation of the Or98a-mediated repellent pathway and pyrethrin-activating Or7a and Or59b pathways was exploited, unknowingly, by humans some thousands of years ago in the formulation of pyrethrum extract as a potent natural insect repellent. We speculate that similar mechanisms might exist for other natural repellents, which are often mixtures of multiple olfactory bioactive components.

Insects respond to volatiles, which often exist as complex mixtures in their environments, by relying on their sophisticated olfactory input and central processing pathways in the peripheral and central nervous systems [36]. Among the pyrethrins-activating Ors, Or7a has previously been shown to be activated by aversive odorants [37,38]. Or7a-expressing ORNs project to the “aversive-specific” glomerulus DL5 in the antennal lobe, whereas Or42b-expressing ORNs activated by attractive odorants innervate the “attractive-specific” glomerulus DM1 [39–42]. Prior to our study, Or59b was shown to be exclusively activated by acetone [37], which elicits attraction in *D*. *melanogaster* [43]. Indeed, we also observed acetone attraction at the 10^−4^ dilution (v v^-1^) (S7 Fig). Furthermore, we found that acetone attraction was abolished in *Or59b*^-/-^ lines, indicating that activation of Or59b mediates attraction (S7 Fig). However, our results also show that pyrethrum repellency is abolished in *Or59b*^-/-^ lines indicating that Or59b has a critical role in pyrethrum repellency. These seemingly contradictory findings may be explained by the differences in the olfactory stimuli: acetone is a single component activating one to a few Ors compared with pyrethrum which is a mixture activating multiple Ors with opposing valences.

EBF is part of herbivore-induced plant volatile blends in tobacco, bean, potato, corn, cotton, sorghum and pine [44–51], providing information on the presence of herbivores. In *Helicoverpa assulta*, EBF activates HassOr23 and one specific glomerulus in the AL and inhibits oviposition of female *H. assulta* in tobacco plants [52]. Aphids release EBF as an alarm pheromone when attacked by predators or parasites [53,54]. In *Acyrthosiphon pisum*, EBF activated ApisOr5 to signal alarm and trigger repellency; and knockdown of the *ApisOr5* transcript by RNA interference abolished the repellency [55]. Repellency of EBF was abolished in the *Or98*^*-/-*^ mutant flies (S8 Fig), demonstrating that EBF activates the Or98a-mediated repellent pathway in *Drosophila*. Recently, we reported that EBF activates Or31 from *Aedes aegypti* and *Anopheles gambiae*. Like Or98a in *D. melanogaster* and *ApisOr5* in *A. pisum* [55], activation of AaOr31 mediates EBF repellency in *Ae. aegypti* [12]. However, there are less than 15% sequence similarities between *AaOr31*, *ApisOr5*, *Or98a* and also *HassOr23* from *H. assulta*. So far, *Or98*, *ApisOr5*, *HassOr23* and *AaOr31* are the only Ors that have been reported to sense EBF.

EBF is a minor component of pyrethrum ranging from 1.25% to 1.97% based on our analysis of the pyrethrum extracts used in this study. At the 10^−4^ dilution (v v^-1^), equivalent to the amount of EBF in our pyrethrum repellency assay in Fig 4, EBF did not elicit repellency (S8 Fig). Therefore, importantly, we have shown that activation of Or98a by EBF in pyrethrum is essential for pyrethrum repellency, even though EBF in pyrethrum by itself is not sufficient to evoke aversion. This suggests that EBF/Or98a contribution to pyrethrum repellency in *Drosophila* depends on pyrethrin-mediated activation of Or7a/Or59b repellency pathways. Notably, not only did the Or98^-/-^ mutant flies lose avoidance response to pyrethrum (Fig 4A), but they also exhibited attraction to pyrethrum (but no attraction to EBF in S8 Fig), highlighting sophisticated interactions between various Or-mediated pathways in response to pyrethrum in determining an ultimate behavioral outcome. The Or98a-mediated repellent pathway could override pyrethrin-activated Or42b-mediated attractive pathway, similar to the geosmin-activated Or56a-mediated repellency, activation of which suppressed attraction by ethyl butyrate [23]. Our findings provide a foundation for further analysis of the neural circuitry that integrates these Or pathways into a potent avoidance response. Future analyses of combinations of double, triple, or quadruple mutants of Or7a, Or42b, Or59b and Or98a would be able to provide further insight into how these Ors interact. In our recent study on the mechanism of pyrethrum repellency in *Ae*. *aegypti*, we found that the low amount of EBF in pyrethrum also makes significant contribution to pyrethrum repellency [12]. Further functional analyses in both insect species could advance our understanding of inter-channel integration in the antennal lobe via lateral connections and/or further integration in the lateral horn [20,56–58].

The two *Drosophila* species examined in this study have very distinct ecological niches. For example, *D*. *suzukii* exhibits stronger attraction to leaf odors than *D*. *melanogaster* in behavioral assays [31,59]. The fact that both *D*. *melanogaster* and *D*. *suzukii* respond similarly to pyrethrum, in electrophysiological and behavioral assays, suggests that the pyrethrum-sensing pathways are conserved between the two species although the mutant systems are not yet available to conduct experiments with the same detail in *D*. *suzukii*. Of note, ORNs activated by pyrethrum are identical between the two species except for ab2B. Pyrethrum activates ab2B neurons in *D*. *suzukii*, but not in *D*. *melanogaster*. Interestingly, Or85a, expressed in ab2B in *D*. *melanogaster*, is lost in *D*. *suzukii* [60]. Conversely, 2-heptanone activates ab2B in *D*. *suzukii* but not in *D*. *melanogaster* [31]. It seems likely that the loss of Or85a is responsible for the change in the response profiles of ab2B in the two species. It is also possible that the differential responsiveness of ab2B to pyrethrum in the two species could be due to the expression of a different (yet to be identified) Or in ab2B of *D*. *suzukii*. Future research should examine how differential activation of ab2B neurons in the two species might influence the integration of neural activities in the central processing of olfactory coding and whether such differential integration contributes to niche-adapted responses to natural odors as well as insect repellents, such as pyrethrum.

*Chrysanthemum* spp. are currently used as companion plants to repel pest insects [9]. Recent studies demonstrated behavioral deterrence of pyrethrin-containing *Chrysanthemum* leaves against western flower thrips (*Frankliniella occidentalis*) [10] and spatial repellency of a pyrethrin precursor against cotton aphids (*Aphis gossypii*) [11]. The Or98a/Or7a/Or59b triple receptors-mediated avoidance mechanism, discovered in this study, could represent an important general olfaction-based strategy for diverse insects to avoid natural insecticidal toxins from plants and for plants to avoid being consumed by insects in a dynamic plant-insect interactive natural world.

## Materials and methods

### Fly stocks

*Drosophila melanogaster* w^1118^ line was used as reference stock, and a *D*. *suzukii* (spotted-wing drosophila) line was field-collected in Michigan in 2016 and maintained in the laboratory since then. Two *Orco* mutants (herein called *Orco*^-/-^ 1*; Orco*^*-/-*^ 2), were obtained from the Bloomington *Drosophila* Stock Center (BDSC) (stock numbers: B23129 and B23130, respectively). The fly lines used in the empty neuron system were kindly provided by John Carlson (Yale University). All flies were raised on BDSC standard cornmeal food: 225 g agar, 2850 g yellow cornmeal, 675 g yeast, 390 g soy flour, 3 L light corn syrup, 39 L water, and 188 ml propionic acid; in an incubator with settings of 25 °C, 60% humidity and a 12 h /12 h day/night light cycle.

### Chemicals

Compounds used for diagnostic stimuli in SSR experiments were from Sigma-Aldrich (Sigma-Aldrich, Milwaukee, including ethyl acetate, ethyl butyrate, 2-heptanone, benzaldehyde, geranyl acetate, pentyl acetate, methyl salicylate, methyl acetate, ethyl hexanoate, E-2-hexenal, geosmin, DEET, 1-octen-3-ol, ethyl lactate, ethyl 3-hydroxybutyrate). They were of the highest grade available (96%-99%). Pyrethrum (Cat# N13151) and (*E*)-β-farnesene (EBF) (purity of 98.5%; Cat# 73492) from Sigma was used in this study. A stock solution of the 10^−1^ dilution (v v^-1^) was made by diluting 200 μL of each compound in 1800 μL of solvent for each compound. Serial decadic dilutions were made from the stock solutions, as needed. Paraffin oil was used as solvent for all electrophysiology recordings, while DMSO was used as solvent for all behavioral assays.

Pyrethrin I, cinerin I, jasmolin I, pyrethrin II, cinerin II and jasmolin II were purified by HPLC with a Shim-pack PREP-SIL silica gel column (20 x 250 mm; Shimadzu) at a flow rate of 10 mL min^-1^ by monitoring the absorbance at 230 nm. As the eluent, a hexane/ethyl acetate mixture (93/7) was used for the purification of pyrethrin I, cinerin I and jasmolin I purification, whereas an 85/15 mixture was used for the purification of pyrethrin II, cinerin II and jasmolin II.

### Behavioral assays

A two-choice assay, as shown in Fig 1A, was modified from the previously described assay [22]. Briefly, to make an assay trap, the tapered end (0.2 cm) of a 1.7 mL microcentrifuge tube (Denville posi-click tubes, Natural color) was cut off; and a 1 mL pipette tip (Tips for Eppendorf Pipettes, Thomas Scientific Inc.) was cut at 2.5 and 0.5 cm from the narrow tip to produce a funnel-like small tip. A 1.6 cm × 1.6 cm filter paper was inserted through the open lid of the cut microcentrifuge tube and secured in by inserting the narrow end of the small tip into the cut microcentrifuge tube (Fig 1A). Fifty microliters of solvent or diluted test compound were applied onto the filter paper and the cut microcentrifuge tubes were then capped. The control and test traps were placed upside-down 2 cm apart in a 100 mL glass beaker and secured using small pieces of double-sided tape. Forty to fifty flies three- to six-day-old flies (both males and females) were gently tapped down from a food vial into the beaker which was already covered with cheese cloth secured with rubber bands. Individual beakers were then placed in individual plastic storage boxes (Snapware, Smart System; 40 cm x 30 cm x 15 cm) into a 25°C incubator. The two-choice attraction assay setup was similar to that of the two-choice assay, except for the addition of 125 μL of 10% apple cider vinegar (ACV) to the upturned lids of cut microcentrifuge tubes as an attractant (S1 Fig).

The T-maze assays was adapted from previously described assay [23] with some modifications (S1 Fig). Briefly, two 1 mL pipette tips (Tips for Eppendorf Pipettes, Thomas Scientific Inc.) and two 1.7 mL microcentrifuge tubes (Denville posi-click tubes, Natural color) were cut and assembled to form two traps. The traps were connected using a 4-cm length and 6.35 mm inner diameter Tygon tubing (Saint-Gobain, Tygon S3 E-3603). Before assembly, a piece of 0.8 cm × 3.2 cm filter paper was lined the wall of the microcentrifuge tube. A 50 μL solvent or test compound of 10^−2^ dilution (v v^-1^) was applied to the filter paper. After the compound was applied, three- to six-day-old *D*. *melanogaster* or *D*. *suzukii* flies (10 males and 10 females) were gently introduced into the Tygon tubing via a third pipette tip which was connected to the tubing via a small hole made in the middle of the tubing. In both microcentrifuge tube lids, a small hole was made to let air flow through.

For all three assays, trials were run for 24 h at 25°C and the number of flies entering each trap was counted. The Repellency Index (RI) was calculated as ((O-C)/(O+C))*100, where O is the number of flies in the test compound trap, C is the number of flies in the control (solvent) trap [22]. The RI ranges from −100% (complete attraction) to 100% (complete avoidance).

### Electroantennography (EAG)

Flies (4–8 days old) were wedged into the narrow end of truncated 200 μL plastic pipette tip and mounted on a microscope slide. The tip of a glass micropipette was used to hold the antenna in a stable position. EAG recordings were conducted as described previously [61]. Reference and recording glass capillary electrodes (1.5 mm outer diameter) were filled with *Drosophila* Ringer’s (in mM): NaCl 100, KCl 5, MgCl_2_ 20, CaCl_2_ 0.15, HEPES 5, sucrose 115, trehalose 5. The reference electrode was inserted into the contralateral eye. The recording electrode was capped onto the anterior distal region of the third antennal segment. The electrodes made electrical contact with a high impedance amplifier (World Precision Instruments, DAM 50) via silver/silver-chloride wires. The signals were digitized with a Digidata 1440A digitizer and Axoscope 10.4 software (Axon Instruments, Molecular Devices). Data were analyzed using Clampfit 10.4 software.

### Single sensillum recording (SSR)

Single sensillum recording was conducted with electrolytically sharpened tungsten microelectrodes as previously described [13,24,31]. A 0.1 mm diameter tungsten wire was sharpened by repeatedly dipping its tip in a 10% KNO_2_ solution electrified at 5–10 mV. Action potentials were recorded by inserting the recording microelectrode in the base of a sensillum, making contact with the lymph surrounding the dendrites of the ORNs. The reference electrode was inserted in the compound eye. The recording electrode was connected to an IDAC-4 signal acquisition system (Syntech, The Netherlands). Signals were fed into a computer and analyzed with Autospike software (Syntech). Signals were counted offline in a 500 ms period before stimulation and for 500 ms during stimulation. Stimulus was controlled using the CS-55 stimulus delivery system (Syntech). Thirty microliters of 10^−2^ dilution (v v^-1^) of odorants or solvent was delivered on a filter paper strip (0.4 cm × 4 cm) which was placed in the shaft of a glass Pasteur pipet serving as an odorant cartridge.

### Gene knockout

Knockout lines were constructed using the CRISPR/Cas9 technology following the method of Gratz *et al*. [62]. Two guide RNAs (gRNAs) for each *Or* were designed by searching the sense and antisense strands of the each ORs gene using the Chopchop (https://chopchop.cbu.uib.no/), CRISPR optical target finder (http://targetfinder.flycrispr.neuro.brown.edu/) and e-CRISPR (http://www.e-crisp.org/E-CRISP/). Sequences of gRNAs were selected based on recommendations by all the three websites for less likely off-target binding. For cloning of gRNA, sense and anti-sense oligos containing the overhang sequences (underlined in S1 Table) to anneal the vector (pU6-BbsI-chiRNA from Addgene), “G” (only for *Or7a*), and CRISPR target sequence were synthesized by Integrated DNA Technologies, Inc. The oligos are phosphorylated using T4 Kinase (Invitrogen) at 37°C for 30min, followed by heating at 95°C for 5 min, then ramp to 25°C at a rate of -0.1°C/sec. for annealing. Annealed oligo was then cloned into the BbsI site of pU6-BbsI-gRNA.

For donor construction for homology-directed repair, 5’ arm and 3’ arm regions of 1Kb upstream and downstream of the CRISPR target site were amplified using Platinum *Taq* DNA Polymerase, High Fidelity (Invitrogen). PCR reaction was heated to 94°C for 2 minutes, followed by 35 cycles of 94°C for 30 seconds, 55°C for 30 seconds, and 68°C for 70 seconds, then 68°C for 7 minutes. PCR product was purified using Wizard SV Gel and PCR Clean-Up System (Promega), then 5’ arm was digested by AarI and cloned into pDSRedattp (Addgene). PCR product of the 3’ arm was then digested with SapI and cloned into the pDSRed-attp with the 5’ arm.

Microinjection, generation, identification of transformants (with DsRed) and balancing were performed by BestGene Inc. Donor plasmid and gRNA plasmids are extracted by QIAGEN Plasmid Midi kit (Qiagen). Each pair of gRNA plasmids and donor plasmid were co-injected into embryos of the transgenic line nanos-Cas9. Deletion of the Ors was confirmed by genomic PCR/sequencing. The sequences of the sgRNAs and details of the knockout lines obtained are summarized in S6 Fig. Primer sequences for PCR and sequencing are summarized in S1 Table. The knockout flies were then back-crossed for at least five generations with the wild-type strain to eliminate potential off-target events.

### Statistical analysis

All statistical analysis was done using SigmaPlot 12.5 (Systat Software). Data are presented as mean ± SEM. Unpaired Student’s *t*-test or Unpaired Mann-Whitney Rank Sum *U*-test (depending on whether assumptions for parametric tests were met) were used to compare results from two treatments. One-Way ANOVA (*F*-test) or One-Way ANOVA on Ranks (Kruskal-Wallis), depending on whether assumptions for parametric tests were met, were used, followed by Dunnett’s test to compare multiple columns of data against a single control. Figures were plotted in SigmaPlot 12.5 and assembled and edited for color and labeling using CorelDRAW Graphic Suit 2020—version 22 (Corel Corporation, Ottawa, Canada).

## Supporting information

S1 Fig Pyrethrum repels *D*. *melanogaster* and *D*. *suzukii*. (A) Schematic drawing of a T-maze assay. (B) T-maze assay measures of pyrethrum repellency in *D*. *melanogaster* [*F*_(2,24)_ = 7.96, *P* = 0.002; *P < 0.05, **P < 0.01 compared to control, One-Way ANOVA, *n* = 8 for control, *n* = 10 for 10^−2^ dilution (v v^-1^), and *n* = 9 for 0.5 dilution (v v^-1^)]. (C) T-maze assay measures of pyrethrum repellency in *D*. *suzukii* [*t* = 4.42, *d*.*f*. = 9, *P* = 0.002; **P < 0.01 compared to control, *t*-test, *n* = 4 for control, and *n* = 7 for 0.5 dilution (v v^-1^)]. (D) Schematic drawing of a two-choice attraction assay. (E) Two-choice attraction assay measures of pyrethrum repellency in *D*. *melanogaster* [*t* = 5.16, *d*.*f*. = 10, *P* < 0.001; ***P < 0.001 compared to control, *t*-test, *n* = 5 for control, and *n* = 7 for 0.5 dilution (v v^-1^)]. (F) Two-choice attraction assay measures of pyrethrum repellency in *D*. *suzukii* [*t* = 10.10, *d*.*f*. = 8, *P* < 0.001; ***P < 0.001 compared to control, *t*-test, *n* = 4 for control, and *n* = 6 for 0.5 dilution (v v^-1^)].
(PDF)

S2 Fig Response profiles of seven types of ab sensilla in *D*. *melanogaster*. Response profiles to a panel of discriminating odorants at 30 μL of the 10^−2^ dilution (v v^-1^) from the seven types of antennal basiconic sensilla (ab1-5 and ab7-8) in *D*. *melanogaster* (*P < 0.05, **P < 0.01, ***P < 0.001, test compound versus control, *n* = 6–10 flies/sensilla).
(PDF)

S3 Fig Response profiles of seven types of ab sensilla in *D*. *suzukii*. Response profiles to a panel of discriminating odorants at 30 μL of the 10^−2^ dilution (v v^-1^) of ab1-5 and ab7-8 in *D*. *suzukii* (*P < 0.05, **P < 0.01, ***P < 0.001, test compound versus control, *n* = 6–10 flies/sensilla).
(PDF)

S4 Fig Representative single sensillum recording traces from ab1-5 and ab7-8 sensilla in response to pyrethrum at 30 μL of the 10^−2^ dilution (v v^-1^) in *D*. *suzukii*. (PDF)

S5 Fig Activation of pyrethrum-responsive Ors by components of pyrethrum in the ab3A empty neuron system. (A) Chemical structures of pyrethrins. (B) Chemical structure of (*E*)-β-farnesene. (C) Representative single sensillum recording (SSR) traces from Or42b, Or59b, Or7a, and Or98a heterologously expressed in the ab3A empty neuron (*ab3*: *Δhalo/Δhalo; Or22a-Gal4/UAS-Orx*) to pyrethrum components (Table 3).
(PDF)

S6 Fig Generation of *Or7a*^*-/-*^, *Or42b*^*-/-*^, *Or59b*^*-/-*^ and *Or98a*^*-/-*^ using the CRISPRCas9 technique. (A) The deletion in Or7a mutants produced a null mutant (0 aa left). (B) The deletion in Or42b mutants produced a severely truncated Or42b protein (N-terminal 18 aa left). (C) The deletion in Or59 mutants produced a null mutant (0 aa left). (D) The deletion in Or98a mutants produced a severely truncated Or98a protein (N-terminal 10 aa left). Genomic region and cytogenetic map (accordingly to https://flybase.org) of each gene is given on top. Solid boxes are exons. Lines are upstream, intron, and downstream DNA sequences. Arrows indicate the sites of target sequences that were used in designing two guide RNAs for CRISPR-Cas9. DNA sequences at these sites are shown below. Start and stop codons are shown in red. PAM motifs are indicated in blue. Sequences underlined are target sequences; lower case letters show DNA sequence. Bold letters show coding regions. The sequence deleted in knockout mutants are indicated in dashed lines. (E-H) Functional validation of knockout of *Or7a*, *Or42b*, *Or59b* and *Or98a*. Representative SSR traces from ab4 (E), ab1 (F), ab2 (G), and ab7 (H) sensilla in Or7a^-/-^, Or42b^-/-^, Or59b^-/-^, and Or98a^-/-^ flies. Upper traces show the absence of response of neurons A to pyrethrum at 30 μL of the 10^−2^ dilution (v v^-1^), and the lower traces show normal responses of corresponding B neurons to their best ligands.
(PDF)

S7 Fig Or59b-mediated acetone attraction in *D*. *melanogaster*. Two-choice assay showing that acetone at 50 μL of the 10^−4^ dilution (v v^-1^) elicits attraction in w^1118^ *D*. *melanogaster* flies. This attraction was abolished in both *Or59b*^-/-^ lines (*H* = 9.28, *d*.*f*. = 2, *P* = 0.01; **P < 0.01 compared to the w^1118^, One-Way ANOVA on Ranks, *n* = 10 for each line).
(PDF)

S8 Fig Behavioral responses of *W*^*1118*^*and Or98a*^*-/-*^ flies to (*E*)-β-farnesene. (A) Two-choice assay to measure fly response to increasing concentrations of (*E*)-β-farnesene [*n* = 13 for 50 μL of the 10^−4^ and 10^−3^ dilutions (v v^-1^), and *n* = 12 for 50 μL of the 10^−2^ dilution (v v^-1^)]. (B) Repellency by (*E*)-β-farnesene was abolished in both *Or98a*^*-/-*^ flies. Behavioral responses of w^1118^ and two Or98a^-/-^ lines to (*E*)-β-farnesene at 10^−2^ dilution (v v^-1^) (*H* = 6.36, *d*.*f*. = 2, *P* < 0.042; *P < 0.05 compared to w^1118^, One-Way ANOVA on Ranks with Dunnett`s test against control, *n* = 16 for each fly line.
(PDF)

S1 Table List of primers used in this study. (PDF)
