# Supplementary material for: Identification of multiple odorant receptors essential for pyrethrum repellency in Drosophila melanogaster
Source: PLoS Genet. 2021 Jul 8;17(7):e1009677. doi: 10.1371/journal.pgen.1009677 (PMC8291717; doi:10.1371/journal.pgen.1009677)
Supplement: S1 Fig — (A) Schematic drawing of a T-maze assay. (B) T-maze assay measures of pyrethrum repellency in D. melanogaster [F(2,24) = 7.96, P = 0.002; *P < 0.05, **P < 0.01 compared to control, One-Way ANOVA, n = 8 for control, n = 10 for 10−2 dilution (v v-1), and n = 9 for 0.5 dilution (v v-1)]. (C) T-maze assay measures of pyrethrum repellency in D. suzukii [t = 4.42, d.f. = 9, P = 0.002; **P < 0.01 compared to control, t-test, n = 4 for control, and n = 7 for 0.5 dilution (v v-1)]. (D) Schematic drawing of a two-choice attraction assay. (E) Two-choice attraction assay measures of pyrethrum repellency in D. melanogaster [t = 5.16, d.f. = 10, P < 0.001; ***P < 0.001 compared to control, t-test, n = 5 for control, and n = 7 for 0.5 dilution (v v-1)]. (F) Two-choice attraction assay measures of pyrethrum repellency in D. suzukii [t = 10.10, d.f. = 8, P < 0.001; ***P < 0.001 compared to control, t-test, n = 4 for control, and n = 6 for 0.5 dilution (v v-1)]. (PDF) [file pgen.1009677.s001.pdf]

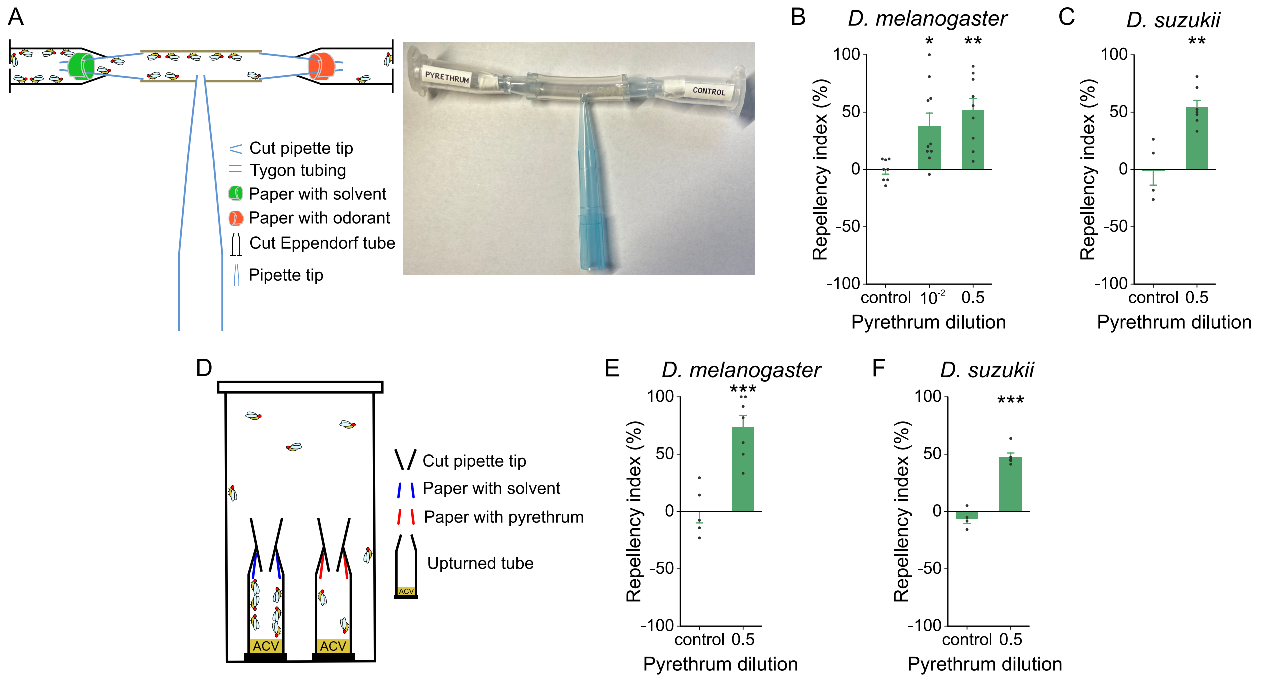

S1 Fig. Pyrethrum repels *D. melanogaster* and *D. suzukii.*

(A) Schematic drawing of a T-maze assay. (B) T-maze assay measures of pyrethrum repellency in *D. melanogaster* [$F_{(2,24)}$ = 7.96, P = 0.002; *P < 0.05, **P < 0.01 compared to control, One-Way ANOVA, n = 8 for control, n = 10 for 10^{-2} dilution (v v^{-1}), and n = 9 for 0.5 dilution (v v^{-1})]. (C) T-maze assay measures of pyrethrum repellency in *D. suzukii* [t = 4.42, *d.f.* = 9, P = 0.002; **P < 0.01 compared to control, *t*-test, n = 4 for control, and n = 7 for 0.5 dilution (v v^{-1})]. (D) Schematic drawing of a two-choice attraction assay. (E) Two-choice attraction assay measures of pyrethrum repellency in *D. melanogaster* [t = 5.16, *d.f.* = 10, P < 0.001; ***P < 0.001 compared to control, *t*-test, n = 5 for control, and n = 7 for 0.5 dilution (v v^{-1})]. (F) Two-choice attraction assay measures of pyrethrum repellency in *D. suzukii* [t = 10.10, *d.f.* = 8, P < 0.001; ***P < 0.001 compared to control, *t*-test, n = 4 for control, and n = 6 for 0.5 dilution (v v^{-1})].
