# Supplementary material for: Identification of multiple odorant receptors essential for pyrethrum repellency in Drosophila melanogaster
Source: PLoS Genet. 2021 Jul 8;17(7):e1009677. doi: 10.1371/journal.pgen.1009677 (PMC8291717; doi:10.1371/journal.pgen.1009677)
Supplement: S2 Fig — Response profiles to a panel of discriminating odorants at 30 μL of the 10−2 dilution (v v-1) from the seven types of antennal basiconic sensilla (ab1-5 and ab7-8) in D. melanogaster (*P < 0.05, **P < 0.01, ***P < 0.001, test compound versus control, n = 6–10 flies/sensilla). (PDF) [file pgen.1009677.s002.pdf]

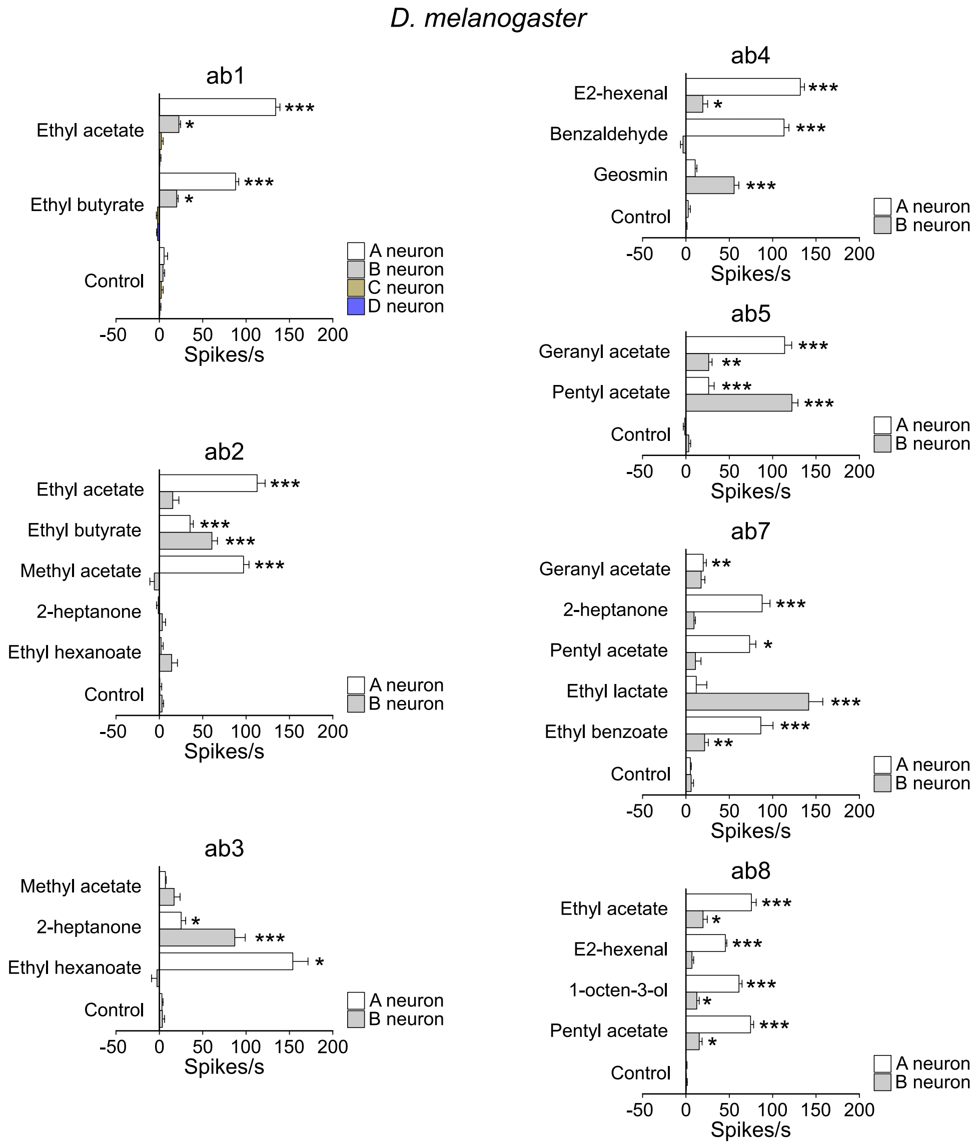

S2 Fig. Response profiles of seven types of ab sensilla in *D. melanogaster*.

Response profiles to a panel of discriminating odorants at 30 μL of the 10^{-2} dilution (v v^{-1}) from the seven types of antennal basiconic sensilla (ab1-5 and ab7-8) in *D. melanogaster* (**P* < 0.05, ***P* < 0.01, ****P* < 0.001, test compound versus control, *n* = 6-10 flies/sensilla).
