# Supplementary material for: Identification of multiple odorant receptors essential for pyrethrum repellency in Drosophila melanogaster
Source: PLoS Genet. 2021 Jul 8;17(7):e1009677. doi: 10.1371/journal.pgen.1009677 (PMC8291717; doi:10.1371/journal.pgen.1009677)
Supplement: S4 Fig — (PDF) [file pgen.1009677.s004.pdf]

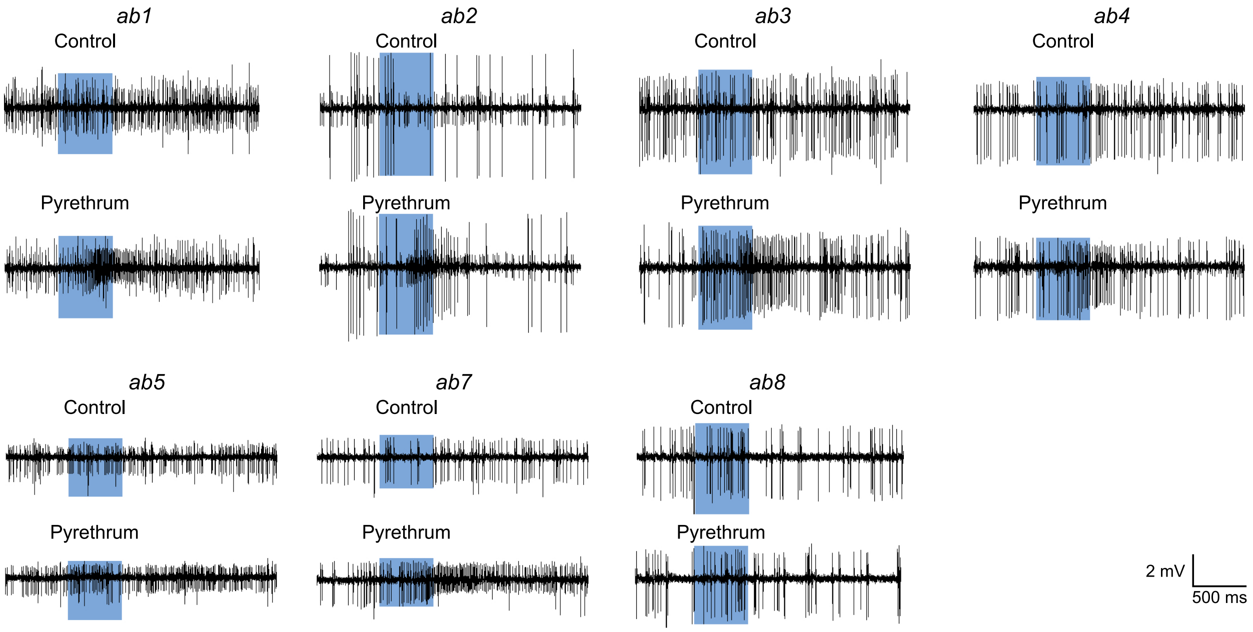

S4 Fig. Representative single sensillum recording traces from ab1-5 and ab7-8 sensilla in response to pyrethrum at 30 μL of the 10^{-2} dilution (v v^{-1}) in *D. suzukii*.
