# Supplementary material for: Identification of multiple odorant receptors essential for pyrethrum repellency in Drosophila melanogaster
Source: PLoS Genet. 2021 Jul 8;17(7):e1009677. doi: 10.1371/journal.pgen.1009677 (PMC8291717; doi:10.1371/journal.pgen.1009677)
Supplement: S5 Fig — (A) Chemical structures of pyrethrins. (B) Chemical structure of (E)-β-farnesene. (C) Representative single sensillum recording (SSR) traces from Or42b, Or59b, Or7a, and Or98a heterologously expressed in the ab3A empty neuron (ab3: Δhalo/Δhalo; Or22a-Gal4/UAS-Orx) to pyrethrum components (Table 3). (PDF) [file pgen.1009677.s005.pdf]

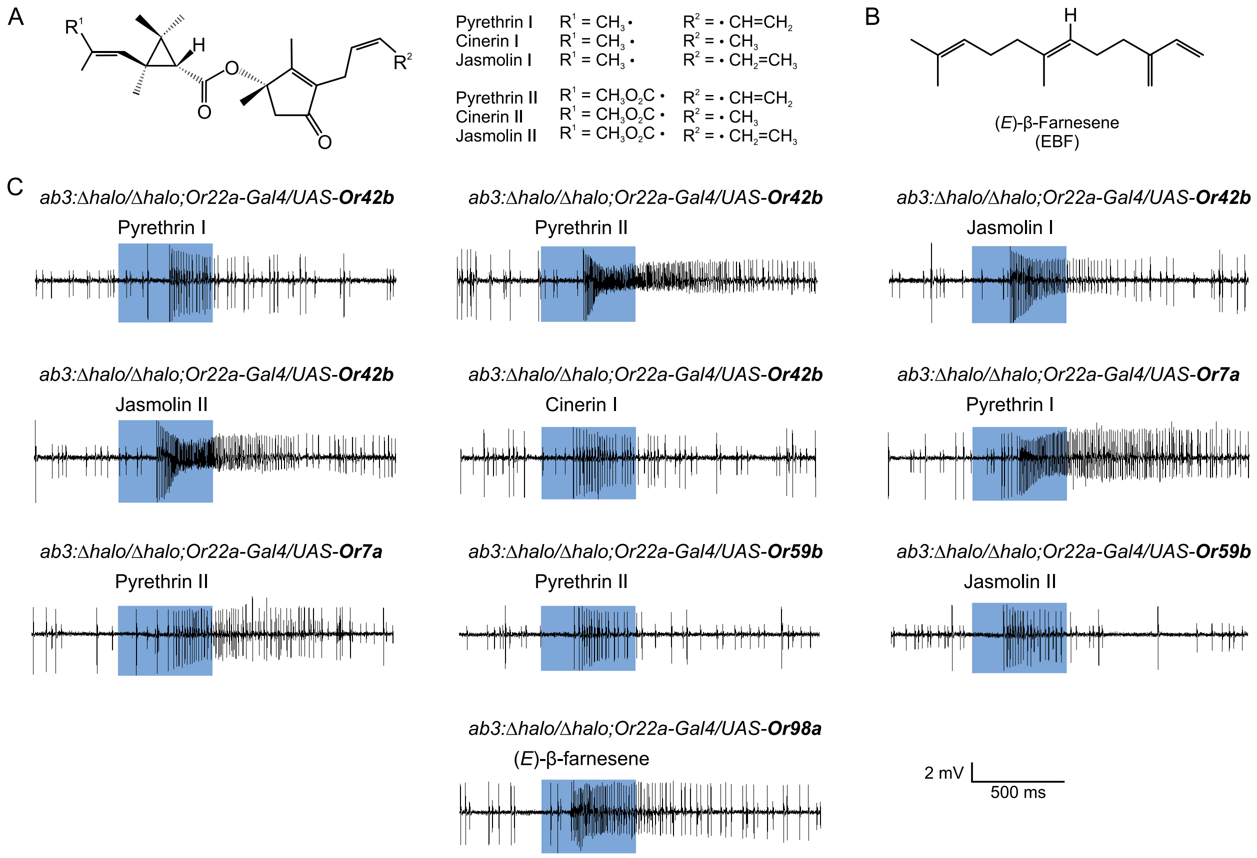

S5 Fig. Activation of pyrethrum-responsive Ors by components of pyrethrum in the ab3A empty neuron system.

(A) Chemical structures of pyrethrins. (B) Chemical structure of (*E*)-β-farnesene. (C) Representative single sensillum recording (SSR) traces from Or42b, Or59b, Or7a, and Or98a heterologously expressed in the ab3A empty neuron (*ab3: Δhalo/Δhalo; Or22a-Gal4/UAS-Orx*) to pyrethrum components (Table 3).
