# Supplementary material for: Identification of multiple odorant receptors essential for pyrethrum repellency in Drosophila melanogaster
Source: PLoS Genet. 2021 Jul 8;17(7):e1009677. doi: 10.1371/journal.pgen.1009677 (PMC8291717; doi:10.1371/journal.pgen.1009677)
Supplement: S6 Fig — (A) The deletion in Or7a mutants produced a null mutant (0 aa left). (B) The deletion in Or42b mutants produced a severely truncated Or42b protein (N-terminal 18 aa left). (C) The deletion in Or59 mutants produced a null mutant (0 aa left). (D) The deletion in Or98a mutants produced a severely truncated Or98a protein (N-terminal 10 aa left). Genomic region and cytogenetic map (accordingly to https://flybase.org) of each gene is given on top. Solid boxes are exons. Lines are upstream, intron, and downstream DNA sequences. Arrows indicate the sites of target sequences that were used in designing two guide RNAs for CRISPR-Cas9. DNA sequences at these sites are shown below. Start and stop codons are shown in red. PAM motifs are indicated in blue. Sequences underlined are target sequences; lower case letters show DNA sequence. Bold letters show coding regions. The sequence deleted in knockout mutants are indicated in dashed lines. (E-H) Functional validation of knockout of Or7a, Or42b, Or59b and Or98a. Representative SSR traces from ab4 (E), ab1 (F), ab2 (G), and ab7 (H) sensilla in Or7a-/-, Or42b-/-, Or59b-/-, and Or98a-/- flies. Upper traces show the absence of response of neurons A to pyrethrum at 30 μL of the 10−2 dilution (v v-1), and the lower traces show normal responses of corresponding B neurons to their best ligands. (PDF) [file pgen.1009677.s006.pdf]

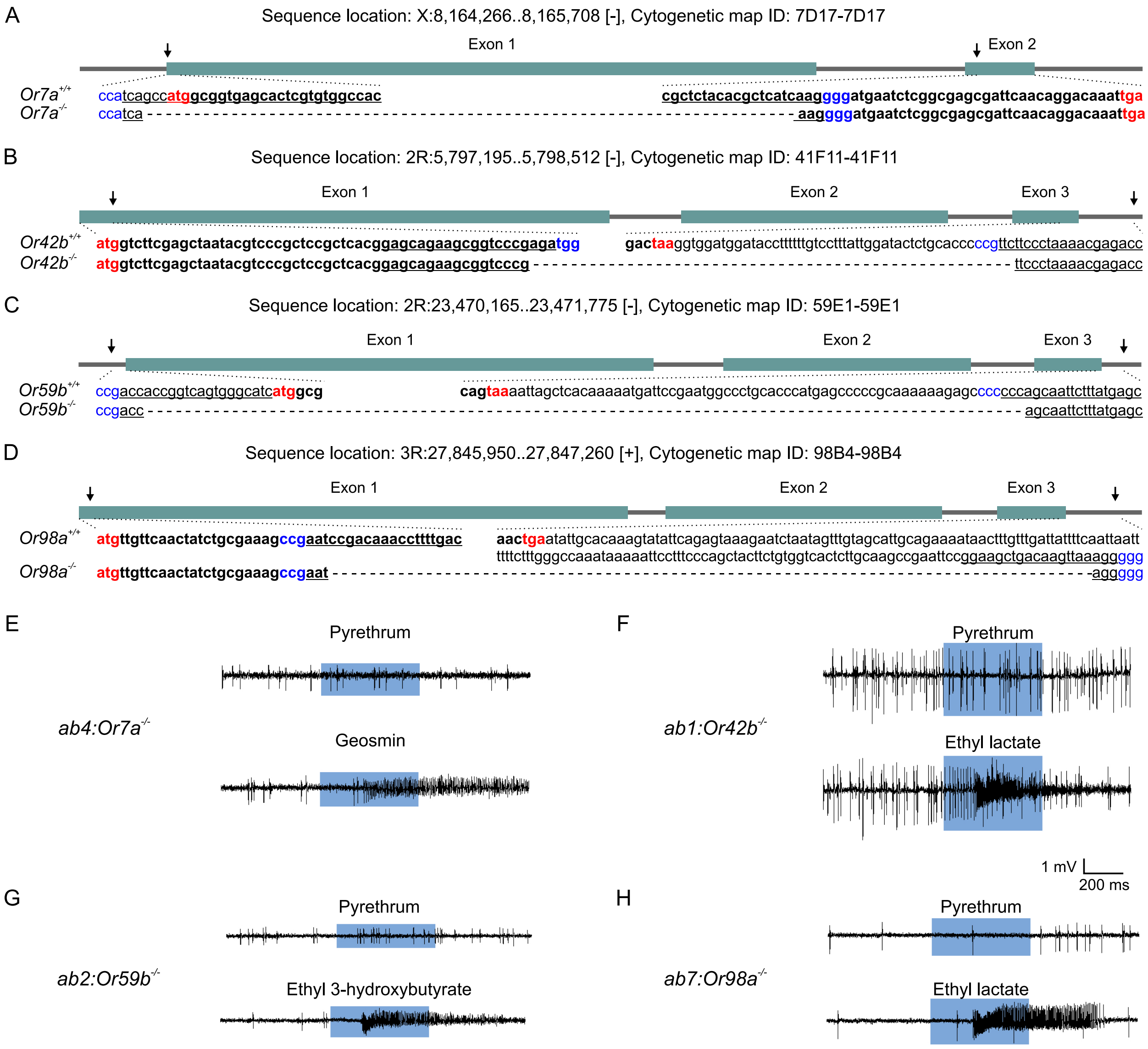

S6 Fig. Generation of *Or7a*$^{-/-}$, *Or42b*$^{-/-}$, *Or59b*$^{-/-}$ and *Or98a*$^{-/-}$ using the CRISPRCas9 technique.

(A) The deletion in Or7a mutants produced a null mutant (0 aa left). (B) The deletion in Or42b mutants produced a severely truncated Or42b protein (N-terminal 18 aa left). (C) The deletion in Or59 mutants produced a null mutant (0 aa left). (D) The deletion in Or98a mutants produced a severely truncated Or98a protein (N-terminal 10 aa left). Genomic region and cytogenetic map (accordingly to https://flybase.org) of each gene is given on top. Solid boxes are exons. Lines are upstream, intron, and downstream DNA sequences.

Arrows indicate the sites of target sequences that were used in designing two guide RNAs for CRISPR-Cas9. DNA sequences at these sites are shown below. Start and stop codons are shown in red. PAM motifs are indicated in blue. Sequences underlined are target sequences; lower case letters show DNA sequence. Bold letters show coding regions. The sequence deleted in knockout mutants are indicated in dashed lines. (E-H) Functional validation of knockout of *Or7a*, *Or42b*, *Or59b* and *Or98a*. Representative SSR traces from ab4 (E), ab1 (F), ab2 (G), and ab7 (H) sensilla in $Or7a^{-/-}$, $Or42b^{-/-}$, $Or59b^{-/-}$, and $Or98a^{-/-}$ flies. Upper traces show the absence of response of neurons A to pyrethrum at 30 μL of the 10^{-2} dilution (v v^{-1}), and the lower traces show normal responses of corresponding B neurons to their best ligands.
