# Supplementary material for: Identification of multiple odorant receptors essential for pyrethrum repellency in Drosophila melanogaster
Source: PLoS Genet. 2021 Jul 8;17(7):e1009677. doi: 10.1371/journal.pgen.1009677 (PMC8291717; doi:10.1371/journal.pgen.1009677)
Supplement: S7 Fig — Two-choice assay showing that acetone at 50 μL of the 10−4 dilution (v v-1) elicits attraction in w1118 D. melanogaster flies. This attraction was abolished in both Or59b-/- lines (H = 9.28, d.f. = 2, P = 0.01; **P < 0.01 compared to the w1118, One-Way ANOVA on Ranks, n = 10 for each line). (PDF) [file pgen.1009677.s007.pdf]

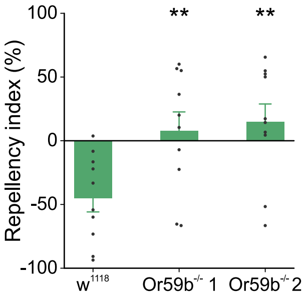

S7 Fig. Or59b-mediated acetone attraction in *D. melanogaster.*

Two-choice assay showing that acetone at 50 µL of the 10^{-4} dilution ($v\ v^{-1}$) elicits attraction in w^{1118} *D. melanogaster* flies. This attraction was abolished in both *Or59b*$^{-/-}$ lines (H = 9.28, *d.f.* = 2, P = 0.01; **$P < 0.01$ compared to the w^{1118}, One-Way ANOVA on Ranks, n = 10 for each line).
