# Supplementary material for: Identification of multiple odorant receptors essential for pyrethrum repellency in Drosophila melanogaster
Source: PLoS Genet. 2021 Jul 8;17(7):e1009677. doi: 10.1371/journal.pgen.1009677 (PMC8291717; doi:10.1371/journal.pgen.1009677)
Supplement: S8 Fig — (A) Two-choice assay to measure fly response to increasing concentrations of (E)-β-farnesene [n = 13 for 50 μL of the 10−4 and 10−3 dilutions (v v-1), and n = 12 for 50 μL of the 10−2 dilution (v v-1)]. (B) Repellency by (E)-β-farnesene was abolished in both Or98a-/- flies. Behavioral responses of w1118 and two Or98a-/- lines to (E)-β-farnesene at 10−2 dilution (v v-1) (H = 6.36, d.f. = 2, P < 0.042; *P < 0.05 compared to w1118, One-Way ANOVA on Ranks with Dunnett`s test against control, n = 16 for each fly line. (PDF) [file pgen.1009677.s008.pdf]

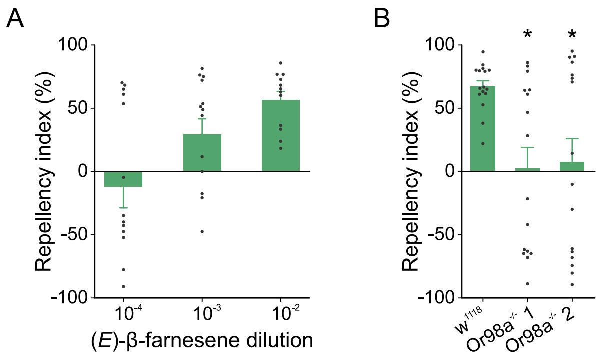

S8 Fig. Behavioral responses of *W^{1118} and Or98a$^{-/-}$* flies to (*E*)-β-farnesene*.*

(A) Two-choice assay to measure fly response to increasing concentrations of (*E*)-β-farnesene [n = 13 for 50 µL of the 10^{-4} and 10^{-3} dilutions (v v^{-1}), and n = 12 for 50 µL of the 10^{-2} dilution (v v^{-1})]. (B) Repellency by (*E*)-β-farnesene was abolished in both *Or98a$^{-/-}$* flies. Behavioral responses of w^{1118} and two $Or98a^{-/-}$ lines to (*E*)-β-farnesene at 10^{-2} dilution (v v^{-1}) (H = 6.36, *d.f.* = 2, P < 0.042; *P < 0.05 compared to w^{1118}, One-Way ANOVA on Ranks with Dunnett`s test against control, n = 16 for each fly line.
