# Supplementary material for: Identification of multiple odorant receptors essential for pyrethrum repellency in Drosophila melanogaster
Source: PLoS Genet. 2021 Jul 8;17(7):e1009677. doi: 10.1371/journal.pgen.1009677 (PMC8291717; doi:10.1371/journal.pgen.1009677)
Supplement: S1 Table — (PDF) [file pgen.1009677.s009.pdf]

S1 Table. List of primers used in this study.

Primer name	Sequence	Used for
Or7a ge F2	GATGGGGTTCTTCGGGTTCTTAAG	genomic PCR & sequencing
Or7a ge R2	ACCACCGAGAAGCAGGCGTAGATG	genomic PCR & sequencing
Or7a ge F3	GAGTTAGAGGATTACCAACTGAGG	genomic PCR & sequencing
Or7a ge R3	GGTTAGCAGGCTTCTGTTCTGGCC	genomic PCR & sequencing
Or7a CRISPR5’chi FW	CTTCGTGCTCACCGCCATGGCTGA	sgRNA
Or7a CRISPR5’chi RV	AAACTCAGCCATGGCGGTGAGCAC	sgRNA
Or7a CRISPR3’chi FW	CTTCGCGCTCTACACGCTCATCAAG	sgRNA
Or7a CRISPR3’chi RV	AAACCTTGATGAGCGTGTAGAGCGC	sgRNA
Or7a 5’arm AarI FW	GGTACACCTGCGCAGTCGCACATAAAGAAGCATATTGCTGCTG	donor
Or7a 5’arm AarI RV	GGACCACCTGCCCTCCTACTGATGGACTTTTGACGCCTGGGAA	donor
Or7a 3’arm SapI FW	GAGTGCTCTTCTTATAAGGGGATGAATCTCGGCGAGCG	donor
Or7a 3’arm SapI RV	GGCTGCTCTTCGGACACAAACTGGATCTGCTGGATGTGG	donor
Or7a ge F4	TAACGAATCGAATCGAAATGAGGG	sequencing
Or7a ge R4	AAAACTAGCGCATCCTATAGATAC	sequencing
Or59b ge 5’F1	AGTTGACAGGGCTGCTTGCACCTC	genomic PCR & sequencing
Or59b ge 5’R1	ACGCAGGTCCAGAAGAGATACACG	genomic PCR & sequencing
Or59b ge 3’F2	CCATTTTAGGTGGCCAAGTTCGCC	genomic PCR & sequencing
Or59b ge 3’R2	GTTGTTACAATCGTCGTCGACTCG	genomic PCR & sequencing
Or59b CRISPR5’chi FW	CTTCGATGCCCACTGACCGGTGGT	sgRNA
Or59b CRISPR5’chi RV	AAACACCACCGGTCAGTGGGCATC	sgRNA
Or59b CRISPR3’chi FW	CTTCGCTCATAAAGAATTGCTGGG	sgRNA
Or59b CRISPR3’chi RV	AAACCCCAGCAATTCTTTATGAGC	sgRNA
Or59b 5’arm AarI FW	GGTACACCTGCGCAGTCGCTCAAGGACGAACCGTTTCAGGTGC	donor
Or59b 5’arm AarI RV	GGACCACCTGCCCTCCTACGGTCGGTGCCAGCAACTGCACTTT	donor
Or59b 3’arm SapI FW	GAGTGCTCTTCTTATAGCAATTCTTTATGAGCGTGTTTTGC	donor
Or59b 3’arm SapI RV	GGCTGCTCTTCGGACCCTTTCCAGCTGCAGCGAAATCAC	donor
Or42b ge 5’F1	TAAACATAACATAGCGCAGCAGAG	genomic PCR & sequencing
Or42b ge 5’R1	ACCACACGAACGTCATTAGCGTCC	genomic PCR & sequencing
Or42b ge 3’F1	CTACCCCATCACTAGGTGGCAAAG	genomic PCR & sequencing
Or42b ge 3’R1	CTAGTATACCCTTTATCTCTACGAG	genomic PCR & sequencing
Or42b CRISPR5’chi FW	CTTCGAGCAGAAGCGGTCCCGAGA	sgRNA
Or42b CRISPR5’chi RV	AAACTCTCGGGACCGCTTCTGCTC	sgRNA
Or42b CRISPR3’chi FW	CTTCGGTCTCGTTTTAGGGAAGAA	sgRNA
Or42b CRISPR3’chi RV	AAACTTCTTCCCTAAAACGAGACC	sgRNA
Or42b 5’arm AarI FW	GGTACACCTGCGCAGTCGCGCTCGGCAGAGTTCTTCGTAGCCG	donor
Or42b 5’arm AarI RV	GGACCACCTGCCCTCCTACCGGGACCGCTTCTGCTCCGTGAGC	donor
Or42b 3’arm SapI FW	GAGTGCTCTTCTTATTTCCCTAAAACGAGACCCCCACAAAC	donor
Or42b 3’arm SapI RV	GGCTGCTCTTCGGACTGTTTGTGGCGATGATCGTCATGG	donor
Or98a 5’ geF1	GATGTGGTTAGCCTAGATTACAGG	genomic PCR & sequencing
Or98a 5’ geRV	TGGCAAGTATACGGCACACCAAGC	genomic PCR & sequencing
Or98a 3’ geF1	TGTTTTTCAGGTGGCTAAGCTGGC	genomic PCR & sequencing
Or98a 3’ geR1	AACTTCCCTTCCAGCAGACAGCCC	genomic PCR & sequencing
Or98a CRISPR5’chi FW	CTTCGTCAAAAGGTTTGTCGGATT	sgRNA
Or98a CRISPR5’chi RV	AAACAATCCGACAAACCTTTTGAC	sgRNA
Or98a CRISPR3’chi FW	CTTCGGAAGCTGACAAGTTAAAGG	sgRNA
Or98a CRISPR3’chi RV	AAACCCTTTAACTTGTCAGCTTCC	sgRNA
Or98a 5’arm AarI FW	GGTACACCTGCGCAGTCGCTTGTGCGTTGCCAGCGTGGGAACG	donor
Or98a 5’arm AarI RV	GGACCACCTGCCCTCCTACATTCGGCTTTCGCAGATAGTTGAAC	donor
Or98a 3’arm SapI FW	GAGTGCTCTTCTTATAGGGGGATTAGAAGCAACTACCAAC	donor
Or98a 3’arm SapI RV	GGCTGCTCTTCGGACAGACAGACAGACTCCTGCATTCGC	donor
